# Sub-district level correlation between tuberculosis notifications and socio-demographic factors in Dhaka City corporation, Bangladesh

**DOI:** 10.1017/S0950268821001679

**Published:** 2021-09-02

**Authors:** Youngji Jo, Yeonsoo Baik, Sourya Shrestha, Jeffrey Pennington, Isabella Gomes, Mehdi Reja, Shamiul Islam, Tapash Roy, Hamidah Hussain, David Dowdy

**Affiliations:** 1 Department of Epidemiology, Johns Hopkins Bloomberg School of Public Health, Baltimore, MD, USA; 2 Challenge TB Project, Interactive Research & Development (IRD), Dhaka, Bangladesh; 3 Interactive Research & Development (IRD), Dhaka, Bangladesh; 4 National Tuberculosis Control Program (NTP), Dhaka, Bangladesh; 5 Interactive Research & Development (IRD) Global, Singapore

**Keywords:** Dhaka, gravity model, sociodemographic risk factors, spatial analysis, tuberculosis

## Abstract

We developed a novel method to align two data sources (TB notifications and the Demographic Health Survey, DHS) captured at different geographic scales. We used this method to identify sociodemographic indicators – specifically population density – that were ecologically correlated with elevated TB notification rates across wards (~100 000 people) in Dhaka, Bangladesh. We found population density was the variable most closely correlated with ward-level TB notification rates (Spearman's rank correlation 0.45). Our approach can be useful, as publicly available data (e.g. DHS data) could help identify factors that are ecologically associated with disease burden when more granular data (e.g. ward-level TB notifications) are not available. Use of this approach might help in designing spatially targeted interventions for TB and other diseases in settings of weak existing data on disease burden at the subdistrict level.

Bangladesh ranks sixth globally in terms of tuberculosis (TB) incidence, with 357 000 estimated cases (221 per 100 000 population) and 80 000 TB deaths in 2018 [1]. While TB incidence is often heterogeneous and influenced by various sociodemographic factors, TB notifications in low- and middle-income countries often lack the specificity sufficient to enable targeting of interventions based on these heterogeneities [2]. Most models, however, use standard TB notification data to estimate the spatial distribution of TB cases in a population. These data may not necessarily reflect where individuals with TB live, where people become infected with TB, or where transmission frequently occurs (e.g. slums, workplaces, public transit, etc.).

Understanding local clustering of TB disease is important to investigate recent TB transmission, especially in high-burden and densely populated urban settings such as Dhaka, Bangladesh. One approach to improving this understanding may utilise the ‘gravity’ model. The gravity model is a widely used formulation of spatial interaction analysis that considers both the distance between two points and their level of ‘attraction’ to each other [3]. In its generic form (describing gravitational attraction between two objects), the gravity model states that the force of attraction between two entities is directly proportional to their masses and inversely proportional to the squared distance separating them. This model has been widely used to explain and understand spatial patterns of population movement including migration, business and commercial travel and the movement of services/information/goods in the form of communication, data transfers or international trade [4]. Several previous studies have applied this model to human health, including primary health services [5] and the spread of diseases including measles, influenza and cholera. [6]

The Bangladesh Demographic and Health Survey (BDHS) is a nationally representative survey with comprehensive sociodemographic information that is easily accessible, but it captures data on a different spatial scale than is used for TB notifications (and likely interventions). At an ecological level, therefore, one could estimate the BDHS data that correspond to TB notification data by first characterising the level of ‘attraction’ between each BDHS area and each TB notification area. Therefore, we aimed to develop a novel use of the gravity model to identify sociodemographic factors collected in the BDHS that might be ecologically associated with TB notifications and therefore useful for spatially targeted TB interventions.

## Setting

Dhaka City Corporation (DCC, population 8.9 million in 2018) consists of 90 wards, divided into Dhaka North City Corporation (DNCC, 36 wards) and Dhaka South City Corporation (DSCC, 54 wards). Wards are the lowest administrative unit for city corporations/municipalities in Bangladesh, with an average population of 100 000. Dhaka has emerged as the fastest-growing and the world's 11th largest city in recent times; about 40% of the city's population lives in 4000 slums and squats [7]. Despite extremely high urban congestion, high living density, socio-spatial divisions, high risk of disease vulnerability, air pollution and unhygienic living conditions, every year, at least 400 000 rural migrants join this city to seek better opportunities of jobs and urban services [8].

## Data sources and study design

We conducted an ecological study to explore geographical correlations between TB incidence and the prevalence of risk factors. We used two publicly available data – national TB notification data and the BDHS data [9].

### National TB notification data

National TB notifications are mandatorily reported via the National Tuberculosis Program (NTP). At the time of this study, an authorised TB reporting centre was located in each ward, the lowest administrative unit in Bangladesh. All people who started treatment in each ward were reported (via a combination of paper-based and electronic records) to the TB reporting centre, which then transmitted an aggregate notification to the NTP on a quarterly basis [10]. In Bangladesh, these notifications include private sector clinics; nevertheless, it is estimated that 30–40% of incident TB cases are not notified – either because the diagnosis is missed entirely, or because it is made in a private clinic that does not interface with the NTP [11]. For this study, we generated estimates of ward-level TB notification rates by aggregating the number of notified TB cases in 2017 (the most recent, available data) within each ward and dividing it by the population size of the ward (assuming 5% annual growth rate in Dhaka city from the 2011 national census). The estimates were then adjusted for observed correlations between TB notifications and actual residence to reflect that 50% of the randomly selected TB cases reported that they resided outside of the ward of their TB diagnosis and treatment. [2]

### Demographic health survey in Bangladesh

Sociodemographic factors including age, sex, education level and wealth index, were extracted from the BDHS conducted in 2013–2014, which was the most recent publicly available data at the time of analysis. The BDHS uses a two-stage stratified sampling design that divides the population of Bangladesh into 600 enumeration areas (EAs, ‘clusters’). EAs represent the geographical units used for BDHS and are larger than wards. While EAs are designed to map onto wards (i.e. each EA should consist of a certain number of wards), this process was imprecise given the rapid changes and urbanisation occurring in Dhaka during this time period. The BDHS sampled 20–30 households per EA, covering all the seven divisions and 64 districts of Bangladesh, based on the 2011 Population and Housing Census – but did not include wards in the sampling frame per se [2]. DCC contains 23 EA-level clusters, including surveys of 610 households and 2641 individuals. Survey weights were applied to address over/under sampling between urban and rural areas and to reflect the representativeness of the country's actual population distribution. The abstracted key sociodemographic factors were re-estimated at the EA-level as the proportion of the population that is male, under 5 years old, over 65 years and having higher than secondary level education. The EA-level wealth index and population density were also provided by BDHS.

## Statistical analyses

To analyse ecological associations between TB notifications (collected at the ward level) and BDHS data (collected at the cluster level), we used a modification of the gravity equation [12], which states that the probability of interaction between two locations is directly proportional to population size and inversely proportional to intervening distance.
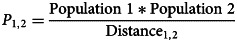
We therefore first calculated the Euclidean distance (in kilometres) between the centroid of each BDHS cluster and the centroid of each ward. We then assigned a weight to each cluster-ward pair based on this distance and the relative population size of each cluster and ward. Using these weights, we calculated the weighted-average BDHS data for each ward (*n* = 90) by multiplying the measured data points for each BDHS cluster (*n* = 23) by the cluster-ward pair weight for each ward (*n* = 23 for each ward), summing these 23 weighted data points for each ward, and dividing this sum by the sum of the 23 weights for that ward. Estimated values were visualised with a choropleth map.

We summarised continuous variables (TB notification rates, proportions of populations under 5 years and over 65 years, population density and household wealth index) using medians and interquartile ranges (IQRs). We used Spearman's rank correlation coefficients to describe the ecological correlation between TB notification rates and sociodemographic factors at the ward level. QGIS version 3.10 was used for mapping and R software version 3.6.2 was used for statistical analyses. (The R Project for Statistical Computing, Vienna, Austria).

## Ethical considerations

All data were de-identified and analysed only in aggregate form. Johns Hopkins School of Public Health Institutional Review Board (IRB) policy does not require IRB oversight for studies involving analysis of de-identified aggregated data.

## Results

**Fig. 1.**
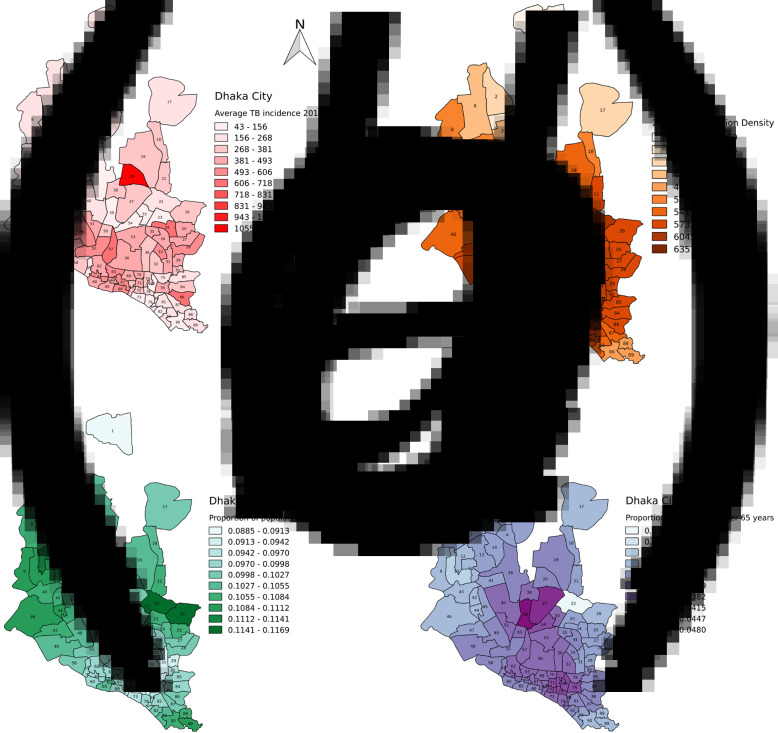
Spatial distribution of TB incidence, population density, proportions of children (age under 5 years) and elderly (age over 65 years). The figure shows the spatial distribution of TB incidence as estimated by TB notification data at the ward level (panel A), and model-based estimates of: population density (panel B), proportions of population under 5 years old (panel C) and over 65 years old (panel D). The latter three measures are based on overlying data from the BDHS (collected across 23 clusters) onto the 90 wards of Dhaka City Corporation, as described in the text. DNCC consists of 36 wards (wards number: 1–23, 37–47, 54–55) and DSCC consists of 54 wards (ward number: 24–36, 48–53,56–90). (a) Average TB notification rate in 2014 and 2017. (b) Population density (population/km^2^) in 2015. (c) % of children (age under 5 years) in 2014. (d) % of elderly (age over 65 years) in 2014.

The spatial distribution of TB notification rates was heterogeneous across wards, ranging from 49 to 1897 per 100 000 per year (Fig. 1), with higher notification rates in DSCC (median: 434, IQR: 277–618) than DNCC (median: 181, IQR: 121–266). Similarly, the model-based estimates of population density and proportion of population over 65 years were greater in DSCC (population density median: 61, 011/km^2^, IQR: 59,470–63,752/km^2^, population over 65 years median: 3.5%, IQR: 3.3–3.6%) than DNCC (population density median: 55 487/km^2^, IQR: 51 425–57 942/km^2^; population over 65 years median: 3.0%, IQR: 2.8–3.2%), whereas the under-5 proportion was greater in DNCC (median: 11%, IQR: 10–11%) than DSCC (median: 9.9%, IQR: 9.6–10%). After using the gravity model to estimate BDHS sociodemographic variables at the ward level, three BDHS variables showed moderate ecological correlations with TB notification rates (Table 1): population density (Spearman's rank correlation 0.45), proportion under 5 years old (−0.34) and proportion over 65 years old (0.28). Population density was the variable most closely correlated with ward-level TB notification rates. Other sociodemographic variables such as gender breakdown, education level and wealth index showed only marginal correlations with TB notification rates.

**Table 1.** Sociodemographic variables from the Bangladesh Demographic Health Survey and their ward-level correlations with incidence of TB

Dhaka City Corporation (Number of wards = 90)	Median (IQR)	Spearman rank correlation with TB notification rate
Average TB notification rate between 2014 and 2017 (per 100 000 per year)^a^	279 (163–425)	–
Population density (persons/km^2^)^b^	59 587 (55 783–61 757)	0.45
Male sex (%)^c^	51 (51–52)	−0.02
Age over 65 years (%)^c^	3.3 (3.0–3.6)	0.28
Age under 5 years (%)^c^	10 (9.7–11.0)	−0.34
Higher than secondary level education (%)^c^	25 (23–26)	0.09
Household wealth index (×100 000)^c^^,^^d^	1.80 (1.74–1.85)	0.17

TB, Tuberculosis.

a Estimated from the national TB notification rate.

b Extracted from the 2015 United Nation population report [2].

c Extracted from the Bangladesh Demographic Health Survey in 2013–2014 [2].

d A standardised score representing a composite measure of a household‘s cumulative living standard calculated on household‘s selected assets.

## Discussion

Our study illustrates the approach of applying a gravity model to overlay two datasets, independently collected at different spatial scales, for purposes of identifying ecological associations between sociodemographic variables and TB notifications in Dhaka, Bangladesh. It is common that different data are collected at different spatial units depending on the purpose of data collection. For example, TB notification data are collected at the ward level reflecting the administrative nature of TB reporting, whereas BDHS data are collected at the EA level as an extension of census data that do not explicitly include wards in the corresponding sampling frame. We used a novel application of the gravity model to identify that population density is moderately correlated with ward-level TB notification rates, suggesting that TB interventions (for example, case finding or preventive therapy) might be more efficiently targeted to wards with higher population density. Our approach can be useful, as publicly available data (e.g. BDHS data) could help identify factors that are ecologically associated with disease burden when more granular data (e.g. ward-level TB notifications) are not available.

Our study focused on the methodological application of the gravity model to identify ecological associations between two independent datasets. Our finding of relatively weak correlation between population-level sociodemographics and TB disease burden may thus partially reflect population mobility, in that ward of notification may not be always same as ward of residence. Although our TB notification rates were adjusted to reflect the discrepancy, the level of discrepancy was not available from all TB cases. As the movement of individuals and reporting patterns could substantially affect the ward-level TB transmission [13], it may be challenging to rely solely on BDHS data or TB notification data to identify areas of intense transmission (i.e. ‘hotspots’). Besides, weak ecological associations between BDHS-measured sociodemographics and TB notifications should not be taken as evidence against the potential for stronger correlations between these characteristics at the level of ward of residence. Future studies could consider collecting additional patient-level data (e.g. reporting and mobility patterns) to better explore the extent to which TB case notifications correlate with ecological-level predictors (e.g. population density or wealth index). These findings could help identify certain wards which may contradict our expectation (e.g. areas with high TB incidence but low population density) and generate new hypotheses (e.g. potential barriers of access to care). They can also guide which additional data to be collected and help develop more effective intervention strategies (e.g. active case finding and household contact investigation in the places with high TB incidence and high proportions of population age under five; active case finding and preventive treatment in the places with high TB incidence and high proportions of population age over 65).

Other studies have used the gravity model to estimate indicators associated with migration between two locations, including human movement patterns [14], and to predict disease spread intensity [6, 12] and healthcare capacity [15]. Here, we illustrate the use of this technique to overlay data collected at different geographic scales for purposes of estimating ecological associations between variables collected in different datasets. Other studies have used statistical techniques to estimate indicators at the local (or small area) level, often by combining survey data with auxiliary data from census or administrative datasets [16]. Since the use of auxiliary information is a key ingredient for successful inference and validation of the model [17], future studies may consider collection or inclusion of other data (e.g. actual population flow between clusters and wards by demographic structure) and incorporate data at different scales (e.g. satellites or individual level survey information) to widen the scope and utility of the application.

Our estimates are consistent with existing knowledge of the demographic and socioeconomic context of the city. Our model-based population density map reflects similar patterns (higher in southwest region in DSCC) compared to other study findings based on original census data [18, 19]. Further information about the location and development of urban slums and informal settlements, as well as the direction or flow of intercity migration (e.g. from north to south), would be helpful to understand how population density might or might not correlate with TB notification rates over time.

The study has several limitations. While we used Euclidean distance and population size as metrics for application of the gravity model, disease transmission is likely to occur on other scales, including dense population clusters (e.g. schools, markets). Future studies could consider alternative formulations of spatial data that incorporate such data. BDHS-derived data likely exhibit some residual spatial clustering across enumeration areas [20], and TB notifications may reflect individuals who live in one ward but are diagnosed and notified in another. Observed correlations for some parameters (such as age distribution compared to population density) may not follow the assumptions of the gravity model (i.e. interactions may follow different distance decay functions or be driven by other exogeneous factors). Like other ecological studies, our estimates are based on observational and aggregate data at the ward level. Therefore, this cannot tell us the transmission pattern of specific age groups, migration between different locations or causal effect of individual or population level socioeconomic characteristics on TB notification. Ecological studies such as this one are subject to both imprecise nature of data at the ecological level and the potential for ecological fallacy (i.e. that associations seen at the ecological level do not hold at the individual level). As such, while useful in describing potential associations between grouped data on sociodemographics (e.g. population density) and TB notification rates, these findings should not be interpreted as supporting a causal or individual-level effect [21]. Future research should consider formally validating these methods, testing other modified gravity model equations (in different geographical settings), and comparing the estimates to other observed data at a subdistrict level. While it was beyond the scope of this analysis, other potential factors (such as mixing, migration or discrepancies in TB case reporting) that may affect these estimates would also be important to consider.

In summary, we used a novel application of the gravity model to identify that population density is moderately correlated with ward-level TB notification rates, suggesting that TB interventions might be more efficiently targeted to wards with higher population density. The method described here can be useful for efficiently utilising available data (e.g. BDHS) for targeted disease control when the units at which interventions might be employed (e.g. wards) do not correspond to the units at which the available data are measured (e.g. BDHS clusters). Use of this approach might help in designing spatially targeted interventions for TB and other diseases in settings of weak existing data on disease burden at the subdistrict level.

## Data Availability

The data that support the findings of this study are openly available in Zenodo open access repository at 10.5281/zenodo.4681050.
